# Keratoameloblastoma of the mandible

**DOI:** 10.4103/0973-029X.72507

**Published:** 2010

**Authors:** BF Adeyemi, AO Adisa, AO Fasola, EE Akang

**Affiliations:** *Department of Oral Pathology, University of Ibadan, Nigeria*; 1 *Department of Oral and Maxillofacial Surgery, University of Ibadan, Nigeria*; 2 *Department of Pathology College of Medicine, University of Ibadan, Nigeria*

**Keywords:** Keratoameloblastoma, mandible, misdiagnosis, prognosis, keratinization, ameloblastoma

## Abstract

Keratoameloblastoma is a very rare ameloblastoma variant defined by extensive squamous metaplasia and keratinization. There are 13 previously reported cases in the literature, with a male predilection of 3:1. A 38-year-old male presented with a painless mandibular swelling which had been progressively increasing in size for 18 months. The incisional biopsy was misdiagnosed as basaloid squamous carcinoma. Owing to financial constraints, the patient had mandibular resection a decade after first noticing the growth, during which the clinical course was essentially benign, thus casting doubt on the initial diagnosis. The final histological diagnosis for both the incisional and resection biopsy specimens was keratoameloblastoma.

## INTRODUCTION

Ameloblastoma is the most common and most intriguing of all odontogenic tumors, though benign.[1,2] It has an unpredictable tendency to metastasize.[3,4] In addition, ameloblastoma is highly polymorphic, due to its ability to undergo various forms of metaplasia. This has given rise to variants of ameloblastoma such as acanthomatous, granular cell, desmoplastic, basal cell, keratoameloblastoma and clear-cell ameloblastoma. The stimulus for the metaplastic change is poorly understood but has been attributed to the multipotentiality of odontogenic epithelium.[2,5]

Keratoameloblastoma, a very rare variant of ameloblastoma with 13 cases described in the literature, was first reported by Pindborg in 1970 and was subsequently described in 1992 as an acanthomatous ameloblastoma with extensive areas of keratinization by the WHO.[6,7] The purpose of this report is to present the histopathological features of a case of keratoameloblastoma seen in our institution and bring to the fore important diagnostic considerations, particularly when handling small biopsy specimens.

## CASE REPORT

On the 26^th^ of April 1999, a 38-year-old Nigerian male presented at our dental clinic for the evaluation of a right mandibular swelling that had been progressively increasing in size for the preceding 18 months, when it was first noticed by the patient. The patient’s chief complaint was a swelling which was asymptomatic. On examination, there was a bony hard swelling extending from the lower right canine to the lower right first molar, with an overlying clinically normal oral mucosa. There was both lingual and buccal expansion of the mandible. Apart from the swelling, the patient appeared clinically healthy. The patient had a full complement of teeth with mobile lower right premolars and lower right first molar. The medical history was insignificant. The clinical differential diagnoses were ossifying fibroma and ameloblastoma. An incisional biopsy was performed, which was reported as basaloid squamous carcinoma.

The financial implication of surgical management made the patient delay treatment for 8 years. During this period, he lost his job as a factory worker, due to irreconcilable social embarrassment secondary to the facial mass.

The patient volunteered that the lower right premolar, lower right first and second molars had exfoliated and the tumor had increased in size. There was an associated history of occasional pain, pus and bloody fluid discharge. The patient had been treated at a private clinic with antibiotics.

On examination, the lesion now extended from the lateral incisor to last molar. It was covered by an erythematous oral mucosa that bled readily on palpation but was not tender on palpation. The swelling measured about 26×20 cm in its greatest diameter. Radiographical examination revealed a multilocular radiolucent lesion which extended from the lateral incisor to the angle of the mandible. Mandibular resection was done with iliac bone reconstruction in April 2008. The surgical procedure was well-tolerated, the post-surgical course was uneventful and no recurrence has been reported.

**Figure 1:**
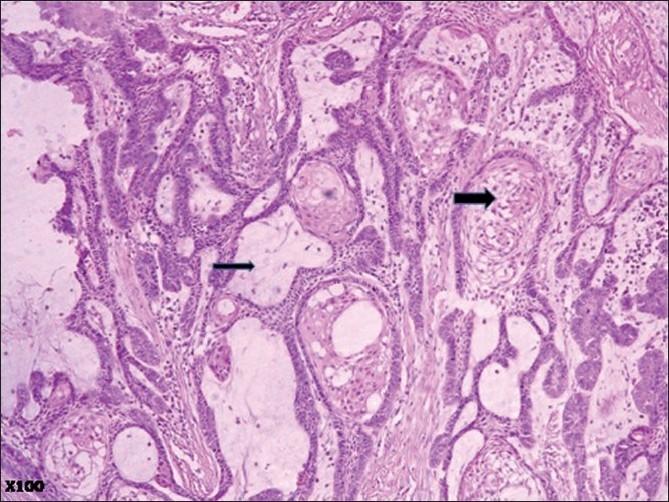
Photomicrograph displaying features of plexiform ameloblastoma with focal areas of squamous metaplasia (thick arrow) and myxoid stroma (thin arrow) (H and E, ×100)

**Figure 2:**
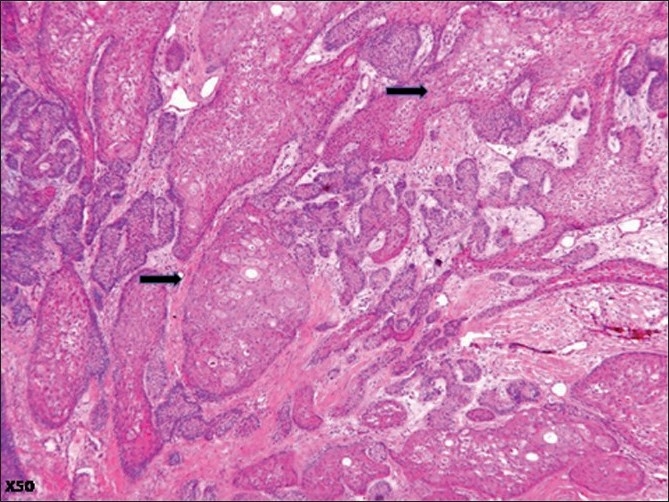
Photomicrograph displaying extensive areas of squamous differentiation within the neoplasm (black arrows) (H and E, ×50)

**Figure 3:**
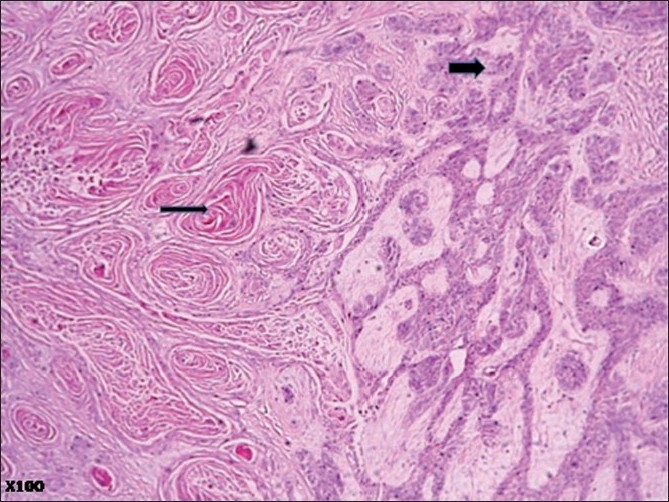
Photomicrograph displaying areas of lamellate parakeratin deposition within the connective tissue stroma, reminiscent of Pacinian corpuscles (thin arrow) and a plexiform pattern (thick arrow) (H and E, ×100)

**Figure 4:**
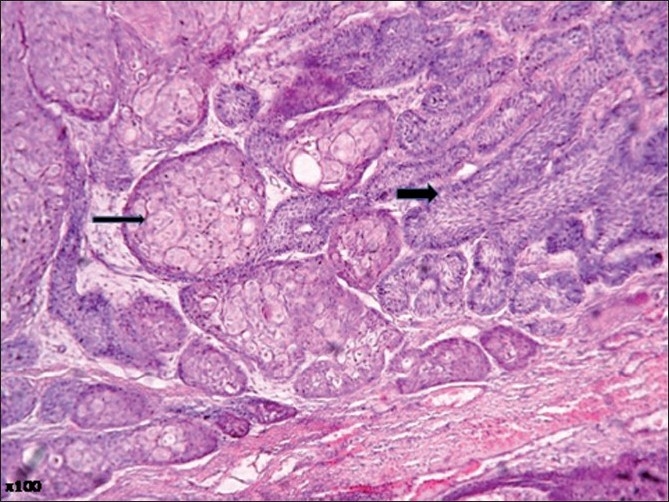
Photomicrograph from original biopsy displaying features consistent with ameloblastoma with plexiform islands of odontogenic epithelium (thick arrow) and focal squamous metaplasia (thin arrow) (H and E, ×100)

Microscopic examination of the surgical specimen showed features of plexiform ameloblastoma [Figure 1] , displaying extensive squamous metaplasia with abundant keratin pearl formation [Figure 2]. In addition, some areas of lamellate parakeratin deposition within the connective tissue stroma displayed an appearance reminiscent of Pacinian corpuscles [Figure 3]. There was no papilliferous component to the neoplasm and no inflammatory reaction to the stromal deposition of parakeratin. There was focal cystic change within the epithelial islands and extensive areas of myxoid stroma change. A diagnosis of keratoameloblastoma was made. Microscopic review of the initial biopsy specimen showed features consistent with this final diagnosis [Figure 4].

## DISCUSSION

The term keratoameloblastoma has been used to describe a heterogeneous group of odontogenic neoplasms, which have in common indisputable histological appearance of ameloblastoma and areas of extensive keratin formation. It differs from acanthomatous ameloblastoma, which also exhibits squamous metaplasia of the stellate reticulum (with or without keratin pearl formation), by the presence of keratin in the connective tissue stroma.[7] The case being presented was diagnosed in a male patient. This is consistent with a male preponderance of 3:1 in reported cases of keratoameloblastoma.[7] In contrast, no gender predilection has been observed in conventional ameloblastoma.[2] Our index case was 38 years old at the time of initial clinical presentation. The majority (38.5%) of the 13 previous cases of keratoameloblastoma were diagnosed in the fourth decade of life, with mean age of 43.8 years while none was diagnosed in the first two decades of life. Keratoameloblastomas are broadly divided into those with and without papilliferous histology.[7] The former tend to present at a relatively older age (mean age 55 years) than the latter (mean age 40 years). Most variants of ameloblastoma have a similar age distribution to keratoameloblastoma, apart from the ameloblastic fibroma and unicystic ameloblastoma, which are more frequently diagnosed in the second decade of life.[1,2]

The present case involved the right mandible which is the site affected in about 54% of reported cases, while the left mandible accounted for 23.1%.[7] Our patient was asymptomatic, apart from the swelling, until the lesion got infected while a few of past reports indicated pain in some of the patients.[7] The index case is distinctive amongst previously reported cases of this rare lesion in that in addition to the well-recognized classical features of keratoameloblastoma, there were extensive areas of myxoid stromal change. Myxoid stromal change has been attributed to the elaboration of heparan sulfate proteoglycan by the tumor cells which is more frequently seen in plexiform histological type of ameloblastoma than follicular pattern.[8]

Three previous reports of keratoameloblastoma that displayed lamellate Pacinian-like stacks of keratin are documented in the English medical literature, making this the fourth case. A case forming hair-like structures has also been reported.[7]

This lesion was previously erroneously diagnosed as basaloid squamous carcinoma. The impression of a malignancy was made due to misinterpretation of the nature of the epithelial islands as malignant basaloid cells with extensive areas of squamous metaplasia. Another reason was our low index of suspicion for this rare lesion. However, the radiographical appearance of the lesion was multilocular, which is very rare in malignant neoplasms. This emphasizes the importance of clinicopathological correlation in histodiagnosis. Basaloid squamous carcinoma is a rare variant of squamous cell carcinoma (SCC) with a predilection for the upper aerodigestive system. Histologically, it is defined as a carcinoma characterized by a basaloid pattern in intimate association with squamous cell carcinoma, dysplasia or focal squamous differentiation.[9,10] It is believed by some authors to be more aggressive than SCC or of same prognosis as poorly differentiated SCC.[9] However, our index case was lost to follow up for about 8 years and showed no clinical sign of malignancy, and thus was treated as a benign lesion, which surgical specimen further proved. Other neoplasms that may pose diagnostic pitfalls include squamous odontogenic tumor and keratinizing ameloblastic carcinoma especially in small biopsy specimens.

Some oral pathologists have speculated that keratoameblastoma may actually be a keratinizing variant of ameloblastic carcinoma.[7] The present case seems to disprove this in that our patient was treated about 10 years after noticing the mandibular growth and the lesion was essentially benign clinically. The clinical behavior of keratoameloblastoma in this case over a period of about a decade, points to the fact that it may not confer a different prognosis from that of conventional ameloblastoma. However, more reports of this very unusual variant of ameloblastoma are encouraged so that its clinical behavior will be well understood.

## CONCLUSION

We have reported a case of keratoameloblastoma whose clinical behavior was similar to that of conventional ameloblastoma. This case highlights the diagnostic challenges associated with small biopsy specimen and the importance of clinicopathological correlation in the diagnosis of these lesions.
